# Dose and route of administration determine the efficacy of prophylactic immunotherapy for peanut allergy in a Brown Norway rat model

**DOI:** 10.3389/fimmu.2023.1121497

**Published:** 2023-02-23

**Authors:** Tiffany Kirkaldy Spaanager Sztuk, Neil Marcus Rigby, Lasse Nørskov-Nielsen, Stef J. Koppelman, Ana Isabel Sancho, Niels-Peter Hell Knudsen, Justin Marsh, Philip Johnson, Shashank Gupta, Alan Robert Mackie, Jeppe Madura Larsen, Katrine Lindholm Bøgh

**Affiliations:** ^1^ National Food Institute, Technical University of Denmark, Kgs. Lyngby, Denmark; ^2^ School of Food Science & Nutrition, University of Leeds, Leeds, United Kingdom; ^3^ Institute of Agriculture and Natural Resources, Food Science and Technology, University of Nebraska-Lincoln, Lincoln, NE, United States; ^4^ Immunology, ALK, Hørsholm, Denmark

**Keywords:** animal model, gastric immunotherapy, oral immunotherapy, subcutaneous immunotherapy, sublingual immunotherapy, food allergy, peanut allergy, IgE

## Abstract

**Introduction:**

Allergen-specific immunotherapy (IT) is emerging as a viable option for treatment of peanut allergy. Yet, prophylactic IT remains unexplored despite early introduction of peanut in infancy was shown to prevent allergy. There is a need to understand how allergens interact with the immune system depending on the route of administration, and how different dosages of allergen may protect from sensitisation and a clinical active allergy. Here we compared peanut allergen delivery *via* the oral, sublingual (SL), intragastric (IG) and subcutaneous (SC) routes for the prevention of peanut allergy in Brown Norway (BN) rats.

**Methods:**

BN rats were administered PBS or three different doses of peanut protein extract (PPE) *via* either oral IT (OIT), SLIT, IGIT or SCIT followed by intraperitoneal (IP) injections of PPE to assess the protection from peanut sensitisation. The development of IgE and IgG1 responses to PPE and the major peanut allergens were evaluated by ELISAs. The clinical response to PPE was assessed by an ear swelling test (EST) and proliferation was assessed by stimulating splenocytes with PPE.

**Results:**

Low and medium dose OIT (1 and 10 mg) and all doses of SCIT (1, 10, 100 µg) induced sensitisation to PPE, whereas high dose OIT (100 mg), SLIT (10, 100 or 1000 µg) or IGIT (1, 10 and 100 mg) did not. High dose OIT and SLIT as well as high and medium dose IGIT prevented sensitisation from the following IP injections of PPE and suppressed PPE-specific IgE levels in a dose-dependent manner. Hence, administration of peanut protein *via* different routes confers different risks for sensitisation and protection from peanut allergy development. Overall, the IgE levels toward the individual major peanut allergens followed the PPE-specific IgE levels.

**Discussion:**

Collectively, this study showed that the preventive effect of allergen-specific IT is determined by the interplay between the specific site of PPE delivery for presentation to the immune system, and the allergen quantity, and that targeting and modulating tolerance mechanisms at specific mucosal sites may be a prophylactic strategy for prevention of peanut allergy.

## 1 Introduction

Food allergy is a disease arising from an inappropriate immune response to otherwise harmless food proteins that causes a clinical reaction upon re-exposure to the offending proteins (1). Food allergic reactions differ in onset and severity depending on the underlying immune mechanism(s) and target organ(s) involved (2), where IgE-mediated food allergies are rapid in clinical onset and can be life-threatening due to the risk of anaphylaxis (3).

Peanut allergy is on the rise (4–8), and is the primary cause of fatal food-induced anaphylaxis in the United States and Western Europe (9–11). Due to the limited availability of effective treatment options and its low resolution rate (12), peanut allergy constitutes a great disease burden (13, 14), where allergen avoidance remains the main viable protective measure against food allergic reactions (15). The constant need for surveillance is associated with anxiety and limits everyday life of peanut allergic patients, which significantly diminishes their quality of life (4, 5, 16). Despite efforts at avoidance, unintentional exposure to peanut is common due to the wide applicability of peanut in both cosmetics and food products (17). Thus, there is an unmet need for effective peanut allergy therapies.

Research efforts within therapies for peanut allergy have pushed allergen-specific immunotherapy (IT) in the forefront as a potential treatment for peanut allergy (3, 18). The fundamental strategy of allergen-specific IT consists of routine administration with allergen for desensitisation and increased reaction threshold to improve quality of life of the peanut allergic patients (19). Strategies under investigation for allergen-specific IT for peanut allergy include different routes of allergen administration such as oral, sublingual (SL) and subcutaneous (SC) allergen administrations, as well as the use of different doses adjusted according to the route of administration (20, 21). The route of administration affects the safety and efficacy profile of allergen-specific IT. Oral IT (OIT) has proven efficacious by increasing reaction threshold, however sustained desensitisation often requires continued exposure to the peanut allergens (22). Furthermore, besides the risk of acute anaphylaxis, many patients experience adverse gastrointestinal symptoms, which is a common cause for ending treatment (23). SLIT may be an alternative to OIT, as the peanut allergen exposure is minute compared to OIT. Indeed, SLIT has been associated with a better safety profile (24), but may be less effective compared to OIT (25). On the other hand, SCIT for peanut allergy has been deemed to be associated with too high a risk of severe side effects to be a viable treatment option (26). These differences may be explained by the different immune responses set off by allergen doses and the site of administration. However, the immunomodulatory effects exerted by different ITs leading to mitigation of peanut allergy is still not fully elucidated and an in-depth comparison of immune profiles set off by a specific dose and route of IT is of high interest to provide a deeper understanding of the safety and efficacy of different ITs.

Given the mortality and morbidity associated with peanut allergy, establishment of allergen-specific IT targeting prevention of peanut allergy are of significant value. The LEAP study assessed whether peanut allergy could be prevented by oral tolerance induction with routine introduction of peanut to the diet of high-risk atopic infants, defined in the LEAP study as infants with severe eczema and/or egg allergy, and showed that early oral introduction had a greater preventive capacity for peanut allergy than peanut allergen avoidance (12). As a consequence, the European Academy of Allergy and Clinical Immunology (EAACI) guideline shifted from recommending allergen avoidance to recommending early oral introduction of peanut as part of complementary feeding for prevention of peanut allergy, with the most appropriate age of introduction suggested to be at four to six months of age (27).

To our knowledge, experimental studies that compare different doses and routes of administration for preventive peanut allergy IT are limited. Therefore, the present study aimed at investigating and comparing how different doses of peanut protein delivered through the oral, SL, intragastric (IG) and SC route of administration affected the primary prevention of peanut allergy in a Brown Norway (BN) rat model of prophylactic IT.

## 2 Materials and methods

### 2.1 Purification of peanut protein extract, Ara h 1, Ara h 2, Ara h 3 and Ara h 6

Peanut protein extract (PPE) was prepared as follows in brief: Raw peanuts were peeled, ground and defatted with hexane (5:1, w/v), re-ground and defatting repeated to obtain defatted peanut flour. Subsequently, peanut proteins from Milli-Q (MQ) water-solubilised peanut flour (10:1, v/w) was collected by repeated centrifugation (3000*g*, 5-20 min, 20°C). The resulting pelleted material was freeze dried and finely ground to produce PPE. Peanut allergens were purified from defatted peanut protein flour obtained from raw peanuts by protein extraction, precipitation and chromatography fractionation, in part based on published methods (28). The detailed methods for PPE preparation and allergen purification are described in the **Supplementary Material**.

### 2.2 SDS-PAGE

The protein profile of PPE, Ara h 1, Ara h 2, Ara h 3 and Ara h 6 was assessed by sodium dodecyl sulfate-polyacrylamide gel electrophoresis (SDS-PAGE) under non-reducing and reducing conditions. Each protein sample was dissolved in PBS (137 mM NaCl, 3 mM KCl, 8 mM Na_2_HPO_4_, 1 mM KH_2_PO_4_, pH 7.2) and mixed 1:1 (v/v) with 2x Laemmli sample buffer (161-0737, Bio-Rad, Hercules, CA, US) with the addition of 2 M dithiothreitol (348-12-3, Sigma-Aldrich, Darmstadt, Germany) for reducing conditions, and subsequently heated for 5 min at 95°C. Five micrograms of protein/well as well as 3 µL of molecular marker (161-0363, Bio-Rad) were loaded onto Mini Protean TGX Precast Protein 4-20% Gels (4568094, Bio-Rad) and electrophoresed on a Mini-PROTEAN Tetra Cell (Bio-Rad) filled with 10X Tris/Glycine/SDS electrophoresis buffer (161-0732, Bio-Rad) (1:10 v/v) prepared according to manufacturer’s protocol. The gels were stained with Coomassie Blue (161-0786, Bio-Rad) at room temperature (RT) for 3 h, and subsequently destained with MQ water at RT overnight. Destained gels were photographed using Imager ChemiDoc XRS+ (Bio-Rad).

### 2.3 Size exclusion chromatography

PPE (1 mg/mL), Ara h 1 (1 mg/mL), Ara h 2 (0.5 mg/mL), Ara h 3 (0.5 mg/mL) and Ara h 6 (0.5 mg/mL) in PBS were filtered through a 0.2 µm filter and individually loaded (50 µL) onto a Superdex 200 Increase 10/300 GL column (Cytiva, Uppsala, Sweden) connected to an ÄKTA pure 25 M system (Cytiva). Each protein sample was eluted at RT with PBS at an elution rate of 0.4 mL/min. The eluted proteins were detected by absorbance at 215 and 280 nm. The column was calibrated for molecular weight (MW) determination by applying a standard mixture consisting of 0.3 mg/mL ferritin (440 kDa; F4503, Sigma-Aldrich), 1 mg/mL conalbumin (79 kDa; C0880, Sigma-Aldrich), 1 mg/mL carbonic anhydrase (29 kDa; C3934, Sigma-Aldrich), 1 mg/mL cytochrome C (14 kDa; C2506, Sigma-Aldrich), 0.5 mg/mL vitamin B12 (1.3 kDa, V2876, Sigma-Aldrich). The presence and approximate quantification of Ara h 1, Ara h 2, Ara h 3 and Ara h 6 present in PPE was determined by overlapping peaks between PPE and the individual allergen followed by calculating area under the curve (AUC).

### 2.4 MS/MS analysis

In brief, PPE was resuspended in PBS, precipitated, reduced, alkylated and digested with trypsin. After C18 clean-up, the resultant peptides were separated by reverse phase HPLC and data-dependent acquisition was carried out by a Q Exactive Plus™ Hybrid Quadrupole-Orbitrap™ MS (Thermo Scientific, Waltham, MA, US). Peaks version 8.5 software was used to process all data-dependent acquisition mass spectral data, using a database of peanut allergens and isoforms [outlined in Apostolovic et al. (29)] and the downloaded reference proteome of peanut. **Supplementary Material** details the methodology.

### 2.5 Animals

BN rats were from the in-house breeding colony (National Food Institute, Technical University of Denmark, Denmark). Rats were kept on an in-house produced diet free from legumes for ≥14 generations to avoid tolerance to proteins homologous to peanut proteins. The diet was as previously described (30), but with maize flakes being substituted with rice flour. Diet and water were given *ad libitum*. Rats were housed in macrolon cages (2-4/cage) under a 12 h light-dark cycle, at 22 ± 1°C and 55 ± 5% relative humidity. Rats were observed twice daily and weighed weekly. Any signs of clinical entities were recorded. Animal experiments were conducted at the National Food Institute facilities and in accordance with Danish legislation; ethical approval was given by the Danish Animal Experiments Inspectorate with the authorisation number 2015-15-0201-00553-C1. The animal experiment was overseen by the National Food Institute’s in-house Animal Welfare Committee for animal care and use.

### 2.6 Animal experimental design

**Figure 1:**
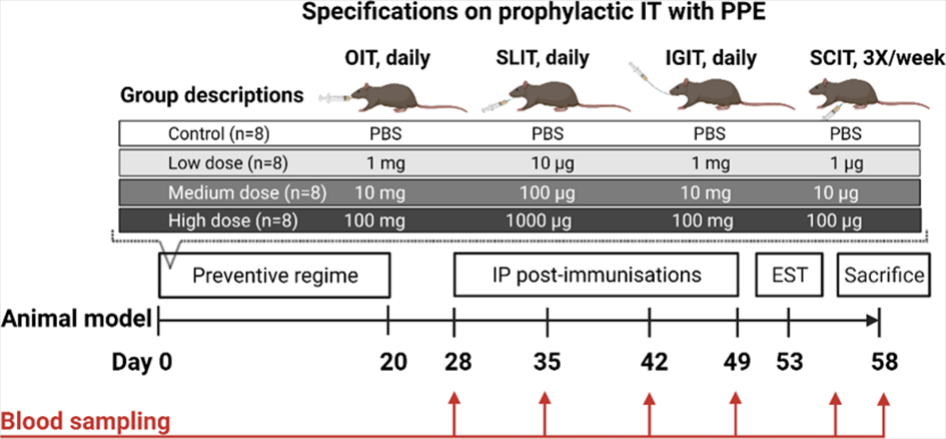
Animal experimental design and specifications on prophylactic immunotherapy (IT) for primary prevention of peanut allergy. Brown Norway (BN) rats (n=8/group) were administered PBS, a low, medium or a high dose of peanut protein extract (PPE) through either the oral (O), sublingual (SL), intragastric (IG) or subcutaneous (SC) route. The preventive regime lasted for three consecutive weeks for each route of administration. To assess the preventive capacity against peanut allergy development induced by administering PPE through the different routes, BN rats were post-immunised intraperitoneally (IP) with 50 µg of PPE every week for four weeks. BN rats were subjected to an ear swelling test (EST) for assessment on clinical peanut allergy. Blood samples were collected throughout the study as indicated. Figure was created with BioRender.com.

BN rats were chosen for the present study as it is an adjuvant free model that allows blood sampling and thorough analyses of antibody-responses on regular basis throughout the experiment. BN rats were allocated into 16 groups (n=8/group, 4/gender, 4-6 weeks of age) and exposed to either a low, medium or high dose of PPE in PBS *via* either oral (1 mg, 10 mg or 100 mg, 0.5 mL daily), SL (10 µg, 100 µg or 1000 µg, 20 µL daily), IG (1 mg, 10 mg or 100 mg, 0.5 mL daily), or SC (1 µg, 10 µg, 100 µg, 250 µL 3X/week) route of administration for three consecutive weeks (Day 0-20). A control group (n=8) receiving PBS was included for each route of administration. Doses were selected based on previous published animal studies (31–33). A description of the execution of oral, SL, IG and SC administrations can be found in the **Supplementary Material**. Rats were post-immunised with 50 µg of PPE in 0.5 mL PBS by intraperitoneal (IP) injection once a week for four consecutive weeks (Day 28, 35, 42 and 49) starting one week after administration of the final preventive dose. After the last IP post-immunisation, BN rats were subjected to an ear swelling test (EST) with 10 µg PPE in 20 µL PBS on Day 53. Blood samples were collected prior to and after the prevention phase (Day 0 and 28), as well as one week after each IP post-immunisation (Day 35, 42, 49 and 56). Rats were euthanised one day after an IP boosting with 1 mg PPE (Day 57) and 30 min after an IG challenge with 100 mg PPE (Day 58) by exsanguination using carbon dioxide inhalation as anesthesia and blood was collected, converted to sera, and stored at -20°C until analysis. Spleens from male rats (n=4/group) were collected upon sacrifice. Only spleens from 4 rats/group were collected to be able to perform *ex vivo* spleen proliferation analyses on individual rats in a single day. Spleens from male rats were chosen as these are much larger than spleens from female rats, and hence allow the harvest of more cells. An outline of the animal experimental design is presented in **Figure 1**.

PPE was tested for endotoxin level by Pierce™ LAL Chromogenic Endotoxin Quantification Kit (A39552, Thermo Scientific) according to manufacturer’s instruction, showing a level of approximately 13 EU per mg of peanut protein.

### 2.7 ELISA – quantification of antibody titres

IgG1 and IgE specific for PPE, Ara h 1, Ara h 2, Ara h 3 and Ara h 6 were quantified by means of indirect and antibody-capture ELISA, respectively. Briefly, IgG1 ELISAs were performed by coating plates (96 well, microtitre, Maxisorp, Nunc, Roskilde, Denmark) with PPE, Ara h 1, Ara h 2, Ara h 3 or Ara h 6 in carbonate buffer (15 mM Na_2_CO_3_, 35 mM NaHCO_3_, pH 9.6), and detection of specific IgG1 was obtained by using horseradish peroxidase (HRP)-labelled mouse-anti-rat-IgG1 (3060-05, Southern Biotech, Birmingham, AL, US). For IgE, ELISA plates (Nunc) were coated with mouse-anti-rat-IgE (HDMAB-123 HybriDomus, Cytotek, Hellebæk, Denmark) and blocked with rabbit serum (S2500-500, Biowest, Nuaillé, France). Specific IgE was detected using digoxigenin (DIG)-coupled PPE, Ara h 1, Ara h 2, Ara h 3 or Ara h 6 and HRP-labelled sheep-anti-DIG (cat. no. 11633716001, Roche Diagnostics GmbH, Mannheim, Germany). A detailed description of the ELISAs is provided in the **Supplementary Material**.

### 2.8 Ear swelling test

BN rats were anesthetised on Day 53 by injection of hypnorm-dormicum and initial ear thickness was measured twice. Subsequently, an EST was performed by intradermally injecting 10 µg of PPE in 20 µL PBS into right ear of each rat as previously described (34). Ear thickness was measured again 30 min after the injection and ear swelling was determined as a measure of the clinical relevance of the peanut sensitisation.

### 2.9 *Ex vivo* spleen proliferation

Spleens (n=4/group) from male control and IT treated rats were removed after sacrifice (Day 58) and placed in sterile PBS for automated tissue dissociation by a gentleMACs dissociator (130-093-235, Miltenyi Biotec, Lund, Sweden) and subsequently centrifuged at 400*g* for 5 min. The pellet was resuspended in PBS and centrifuged again at 400*g* for 5 min. The resulting pellet was suspended in 5 mL RPMI 1640 medium (72400-021, Gibco, Brazil) with 5% (v/v) FBS (10500-064, Gibco), 0.1% (v/v) [50 µg/mL] Gentamycin (15750-037, Gibco), and 0.15 µM [16.6 µg/mL] monothioglycerol (M6145, Sigma-Aldrich). Cells were counted and added to 96-well-flat-bottom culture plates (Costar, REF 3917, Merck Life Science, Søborg, Denmark). Stimulation of splenocytes was performed by adding 50 µg PPE to each well. The number of live splenocytes was determined by absorbance quantification measured by Glomax proliferation CellTiter-Glo (Promega, Madison, US). See **Supplementary Material** for a detailed description.

### 2.10 Statistical analyses

Graphs and statistical analyses were performed in Prism version 9.3.1 (Graphpad, San Diego, CA, US). Results were analysed using non-parameteric statistical tests, as data in experimental groups were not normal distributed assessed by D’Agostino-Pearson normality test. Kruskal-Wallis test followed by Dunn’s post-test was performed for analysing multiple comparisons between medians from more than two groups. To determine the significance between the association of dose and the level of IgG1 or IgE, as well as between IgE and ear swelling response to PPE, the non-parametric Spearman’s correlation was applied. Control animals were only included in the non-parametric Spearman correlations for ELISA results on day of sacrifice (Day 58). Asterisk(s) indicate statistically significant difference between two given groups: *p ≤ 0.05, **p ≤ 0.01 and ***p ≤ 0.001, ****p ≤ 0.0001.

## 3 Results

### 3.1 PPE contains all major peanut allergens

**Figure 2:**
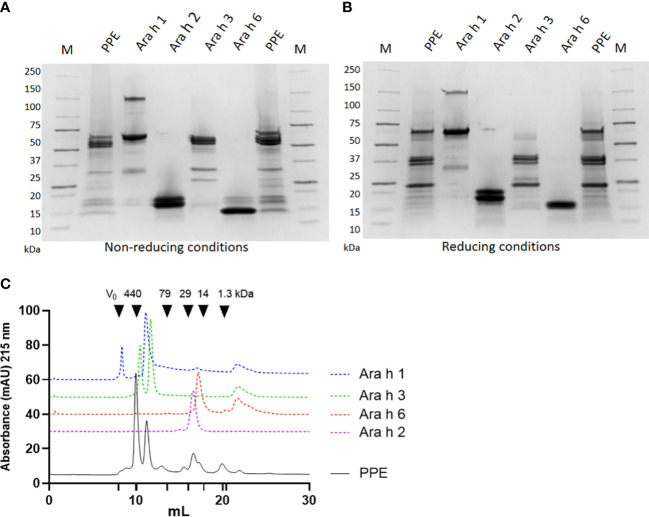
Allergen profile of peanut protein extract (PPE). The protein profile of PPE, Ara h 1, Ara h 2, Ara h 3 and Ara h 6 was analysed by sodium sulfate-polyacrylamide gel electrophoresis (SDS-PAGE) under **(A)** non-reducing and **(B)** reducing conditions followed by Coomassie staining. M indicates molecular weight marker. **(C)** PPE and Ara h 1, Ara h 2, Ara h 3 and Ara h 6 were analysed by size exclusion chromatography at 215 nm. Chromatograms of Ara h 1, Ara h 2, Ara h 3 and Ara h 6 are shifted vertically compared to the chromatogram of PPE, to improve readability.

PPE was prepared from whole peanut and analysed for the presence of known peanut allergens before use in animal experiments. The protein profile analysed by SDS-PAGE under both non-reducing and reducing conditions suggested the presence of all major peanut allergens (Ara h 1, Ara h 2, Ara h 3 and Ara h 6), as bands with comparable migration patterns to individual purified peanut allergens were present in PPE (**Figures 2A, B**). The SDS-PAGE revealed that Ara h 3 was present in a higher amount than Ara h 1, Ara h 2 and Ara h 6. Under both non-reducing and reducing conditions Ara h 1 showed a major band around 65 kDa corresponding to its monomeric form, and a band of about 150 kDa likely corresponding to its trimeric form. The migration pattern of Ara h 2 was similar under non-reducing and reducing conditions, showing two individual bands of 17 and 19 kDa likely corresponding to the Ara h 2.01 and Ara h 2.02 isoforms, respectively. Contrary, Ara h 3 showed different patterns of migration under non-reducing and reducing conditions. Under reduced conditions the Ara h 3 protein was separated into its subunits with separate bands of around 37 kDa and 25 kDa, whereas under non-reduced conditions Ara h 3 showed two major bands of around 55-60 kDa, corresponding to the basic and acidic subunits linked together as a single protein. Ara h 6 showed a major band of around 15 kDa under both non-reducing and reducing conditions, with additional bands of around 17 and 19 kDa with low intensity only present under non-reducing conditions.

Quantification of the major allergens in PPE was performed by size exclusion chromatography (SEC). Guided by chromatograms of the individual allergens, the AUC of the individual Ara h allergen peaks was determined in PPE (**Figure 2C**). This analysis revealed that Ara h 3 was the most abundant allergen in PPE constituting approximately 45% of the protein content, and that Ara h 1, Ara h 2 and Ara h 6 were present in lower but comparable amounts constituting each approximately 10%. This corresponded well with the protein profile revealed by SDS-PAGE (**Figures 2A, B**). MS/MS analysis confirmed the presence of the four major peanut allergens and identified the presence of Ara h 1 isotypes, Ara h 2 isotypes, Ara h 3 isotypes, Ara h 6 isotypes and Ara h 7 isotypes (**Supplementary Material Table S1**). The mass spectrometry proteomics data have been deposited to the ProteomeXchange Consortium *via* the PRIDE (35) partner repository with the dataset identifier PXD039324 and 10.6019/PXD039324.

### 3.2 Preventive OIT and SCIT, but not IGIT or SLIT, induce PPE-specific IgG1

**Figure 3:**
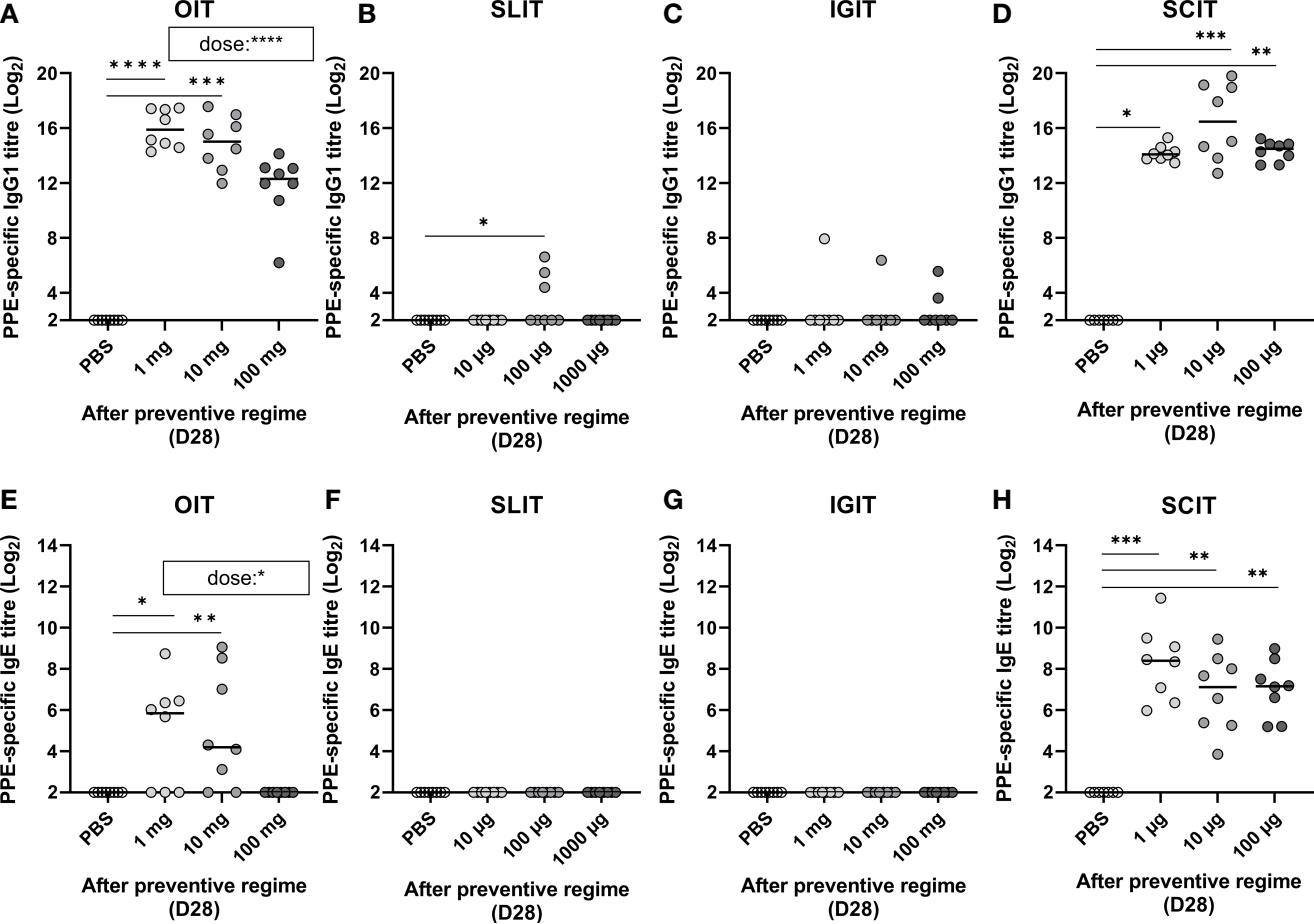
Peanut protein extract (PPE)-specific IgG1 and IgE in response to dose and route of prophylactic immunotherapy (IT) on Day 28 (D28). Antibody titres for PPE-specific IgG1 and IgE present in animal sera determined by ELISA after exposure to three weeks of preventive regime (Day 28) with **(A, E)** oral (O)IT, **(B, F)** sublingual (SL)IT, **(C, G)** intragastric (IG)IT and **(D, H)** subcutaneous (SC)IT. Each symbol represents a single animal and horizontal lines indicate the median of each group. Statistically significant differences compared to control animals receiving PBS administration are indicated with asterisk(s), * p<0.05, ** p<0.01, *** p<0.001, **** p<0.0001. A statistically significant correlation (p value noted in brackets on **(A, E)**) between PPE dose and PPE IgG1 and IgE was exclusively evident for OIT.

PPE was administered for three consecutive weeks by means of OIT, SLIT, IGIT, or SCIT in peanut naïve animals to compare preventive capacity against peanut allergy. PPE-specific IgG1 levels were assessed by ELISA after the preventive regime (Day 28). All doses of PPE in OIT induced high levels of IgG1 in a dose-dependent manner with the low and medium dose administered animals being statistically significant different to control animals receiving PBS (**Figures 3A**). Contrary, only a few animals receiving PPE *via* SLIT or IGIT developed detectable levels of PPE-specific IgG1 after the prevention regime (**Figures 3B, C**). All animals administered with PPE *via* SCIT, irrespective of the dose, developed statistically significant levels of PPE-specific IgG1 compared to control animals receiving PBS (**Figure 3D**). Collectively, these results indicate that PPE predominantly induce an IgG1-related immune response when administrated *via* the oral and SC routes, but not *via* SL or IG routes.

### 3.3 Preventive OIT and SCIT, but not IGIT or SLIT, induce sensitisation to peanut

PPE-specific IgE was analysed to assess if PPE administration induced sensitisation after the 3 weeks of prophylactic OIT, SLIT, IGIT or SCIT (Day 28). OIT was found to induce sensitisation in a dose-dependent manner, with the lowest dose (1 mg) giving rise to the highest levels of PPE-specific IgE (**Figure 3E**). Contrary, none of the animals in the highest OIT group (100 mg) became sensitised to PPE. SLIT or IGIT did not induce sensitisation, as no PPE-specific IgE could be detected after administration with any of the PPE doses in the preventive regimes (**Figures 3F, G**). In contrast to OIT, SLIT and IGIT, all doses of SCIT induced PPE-specific IgE, with all groups of PPE dosed animals showing statistically significant differences to control animals administered with PBS (**Figure 3H**). Additionally, PPE *via* SCIT administration promoted a higher level of PPE-specific IgE than any other IT, irrespective of the doses applied. Collectively, these results indicate that PPE can induce sensitisation when administrated *via* oral and SC route, but not *via* SL or IG route, in peanut naïve animals.

### 3.4 OIT, SLIT, and IGIT show different dose-dependent prevention of PPE sensitisation

**Figure 4:**
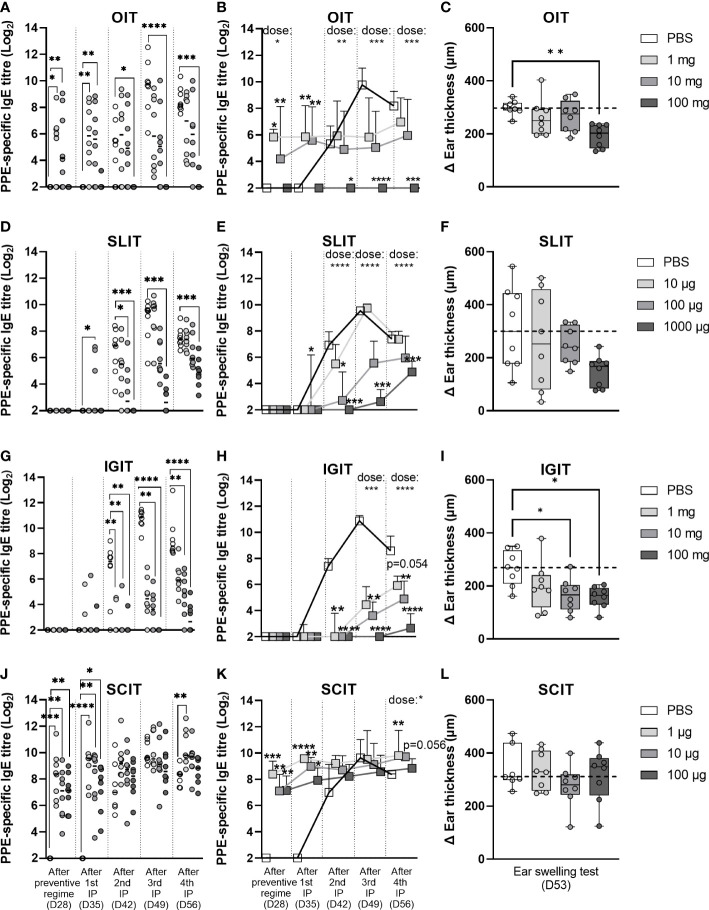
Prevention of peanut protein extract (PPE)-induced sensitisation and clinical response. Prevention of intraperitoneally (IP)-induced sensitisation was analysed by determining the levels of PPE-specific IgE present in rat sera after preventive regime with **(A, B)** oral immunotherapy (OIT), **(D, E)** sublingual (SL)IT, **(G, H)** intragastric (IG)IT or **(J, K)** subcutaneous (SC)IT, as well as after each IP post-immunisation. Each circle in the left panel **(A, D, G, J)** depicts the level of PPE-specific IgE of an individual animal and horizontal lines indicate median values in each group. The order of groups from left to right is for all days and all preventive regimes; PBS, low dose, medium dose and high dose. Square symbols in the middle panel **(B, E, H, K)** represent the median value + upper interquartile range in each group (n=7/8) and are connected by straight lines from day to day to illustrate the progression of PPE-specific IgE during the time course of the animal experiment. Prevention of PPE-induced elicitation was assessed by an ear swelling test with 10 µg of PPE intradermally injected into the right ear of each rat and is illustrated in the right panel **(C, F, I, L)** with each individual animal presented by a circle. Ear thickness was measured prior to injection and 30 min after injection with PPE, thus delta ear thickness indicates the increase in ear thickness in response to PPE. Statistically significant differences compared to control animals receiving PBS administration are indicated with asterisk(s), * p<0.05, ** p<0.01, *** p<0.001, **** p<0.0001.

To assess the efficacy of the three weeks of prophylactic OIT, SLIT, IGIT or SCIT regimes in preventing peanut sensitisation, animals were post-immunised with weekly IP injections of PPE for four weeks (Day 28, 35, 42 and 49). Sensitisation was evaluated by analyses of PPE-specific IgE levels after the prevention regimen (Day 28), and after first (Day 35), second (Day 42), third (Day 49) and fourth (Day 56) post-immunisation by ELISA. Control animals (PBS) became readily sensitised and developed increasing levels of PPE-specific IgE detectable after the second PPE IP injection (**Figure 4**). Control animals likewise developed increasing levels of PPE-specific IgG1 after the second IP injection with PPE (**Supplementary Material Figure S1A-D**).

PPE OIT was found to suppress IP-induced sensitisation to PPE in a dose-dependent manner, though only the high dose (100 mg) PPE OIT was found to prevent IP-induced sensitisation to PPE to a statistically significant degree (**Figures 4A, B**). This preventive effect was also associated with a statistically lower clinical response to PPE as demonstrated by the EST (**Figure 4C**). The low and medium doses of PPE OIT, which induced sensitisation during the preventive regime, were unable to prevent IP-induced sensitisation to PPE to a statistically significant degree.

PPE SLIT was also found to suppress IP-induced sensitisation to PPE in a dose-dependent manner (**Figures 4D, E**). PPE administration suppressed the levels of both PPE-specific IgG1 (**Supplementary Material Figure S1B**) and IgE (**Figure 4E**) after the second, third and fourth IP injection. Clinical reactivity to PPE also followed a dose-dependent pattern as indicated by the EST results (**Figure 4F**), although no doses showed statistically significant protection from PPE-induced elicitation (p = 0.08 for 1000 µg PPE SLIT).

PPE IGIT was likewise found to suppress IP-induced sensitisation in a dose-dependent manner. The administered PPE dose correlated inversely with the level of PPE-specific IgE after the second IP injection with PPE (**Figures 4G, H**). The suppression of IP-induced PPE-specific IgE was associated with a lower clinical reactivity to PPE, as demonstrated by the EST (**Figure 4I**). A correlation between the dose of PPE and the level of PPE-specific IgG1 was observed after the third and fourth IP injection (**Supplementary Material Figure S1C**).

PPE SCIT showed no preventive effect for IP-induced sensitisation with all groups showing comparable levels of PPE-specific IgE in serum (**Figures 4J, K**) and PPE-induced elicitation after EST (**Figure 4L**). This was not surprising as all animals, irrespective of PPE dose, were sensitised after the three weeks of preventive SCIT regimen.

Overall, high doses of PPE *via* oral and SL route as well as medium and high doses of PPE *via* IG route suppressed IP-induced sensitisation. These preventive effects for IP-induced sensitisation were also reflected in the level of protection of PPE-induced elicitation after EST, where a significant correlation between IgE at Day 56 and ear swelling was observed (**Supplementary Material Figure S2**).

### 3.5 OIT, SLIT and IGIT show similar preventive effect for sensitisation towards individual peanut allergens

PPE, Ara h 1, Ara h 2, Ara h 3 and Ara h 6-specific IgG1 and IgE ELISAs were performed with sera from the day of sacrifice (Day 58) in order to determine the capacity of OIT, SLIT, IGIT and SCIT in preventing sensitisation to the individual major peanut allergens.

**Figure 5:**
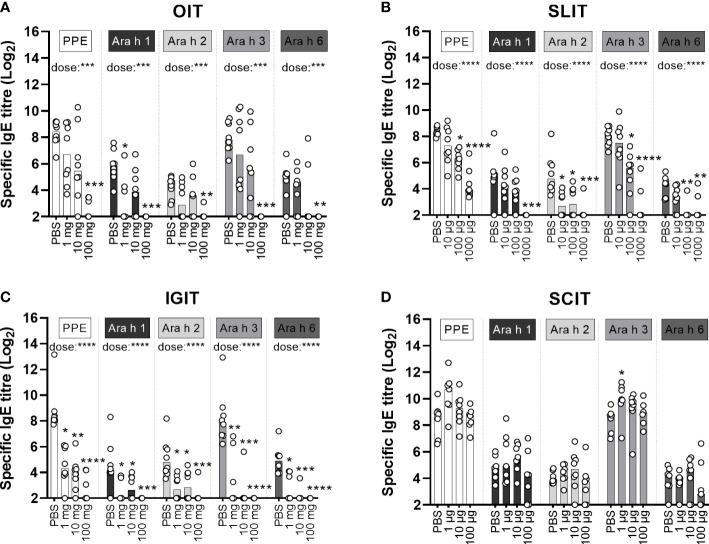
Allergen-specific IgE analyses. Levels of peanut protein extract (PPE), Ara h 1, Ara h 2, Ara h 3 and Ara h 6-specific IgE were quantified by ELISA in sera from the day of sacrifice (D58). Median bars are presented for each group and each circle represents a single animal. Each specificity of IgE demonstrates the specific level of sensitisation and is the sum of a three week’s preventive regime (Day 0-20) with **(A)** oral immunotherapy (OIT), **(B)** sublingual (SL)IT, **(C)** intragastric (IG)IT or **(D)** subcutaneous (SC)IT with three different doses of PPE or PBS followed by four intraperitoneal (IP) injections with PPE (Day 28, 35, 42 and 49) for assessing the efficacy of each IT. Statistically significant differences compared to PBS administration are indicated with asterisk(s), * p<0.05, ** p<0.01, *** p<0.001, **** p<0.0001.

Allergen-specific IgG1 and IgE analyses of control animals administered with PBS during the preventive regimen showed that IP post-immunisations induced significant levels of both Ara h 1, Ara h 2, Ara h 3 and Ara h 6 specific-IgG1 and IgE in animals on Day 58 (**Figures 5A–D**; **Supplementary Materials S3A-D**). This demonstrates that the animals recognised all major peanut allergens and could be sensitised to all of them upon IP injections regardless of their different proportions in PPE. Although a direct comparison between assays cannot be made, the allergen-specific IgG1 analyses suggested that Ara h 1 induced the lowest IgG1 response (**Supplementary Material Figures S3A-D**), and the allergen-specific IgE analyses suggested that Ara h 3 induced the highest level of specific IgE (**Figures 5A–D**), which may be expected as Ara h 3 was the most abundant allergen in PPE.

IgE and IgG1 levels toward the major allergens were suppressed by PPE OIT in a dose-dependent manner (**Figure 5A**; **Supplementary Material Figure S3A**). Yet, only the high dose PPE OIT prevented the development of PPE-specific IgE, and hence sensitisation to all major allergens (**Figure 5A**).

Overall, IgG1 and IgE levels toward the major allergens were suppressed by PPE SLIT in a dose-dependent manner (**Figure 5B**; **Supplementary Material Figure S3B**). Medium and high dose PPE SLIT suppressed the development of PPE-specific IgE compared to control animals administered with PBS (**Figure 5B**). The same pattern was reflected in the major allergen-specific IgE levels, with the exception of prevention for sensitisation to Ara h 1 that was only suppressed by high dose PPE SLIT (**Figure 5B**). Contrary, all doses of SLIT reduced Ara h 2-specific IgE levels (**Figure 5B**).

Similar to OIT and SLIT, both IgG1 and IgE levels toward the major allergens were suppressed by PPE IGIT in a dose-dependent manner (**Figure 5C**; **Supplementary Material Figure S3C**). In addition, all doses of PPE IGIT suppressed the development of PPE-specific IgE compared to PBS administration, and hence sensitisation to all major allergens, indicating that prophylactic IGIT constitutes a very efficient route of PPE administration for prevention of peanut allergy (**Figure 5C**).

In contrast, none of the doses of PPE SCIT prevented sensitisation to PPE compared to control PBS and likewise did not prevent the sensitisation to any of the major allergens (**Figure 5D**).

In summary, the pattern of sensitisation and levels of IgE toward the major peanut allergens overall followed the PPE sensitisation and PPE-specific IgE levels, with suppression of IgE in a dose-dependent manner for PPE administrated *via* OIT, SLIT and IGIT. These findings indicate that no specific major allergen in PPE drives the sensitising or preventive capacity of PPE.

### 3.6 OIT and IGIT suppress the *ex vivo* proliferative response to PPE

**Figure 6:**
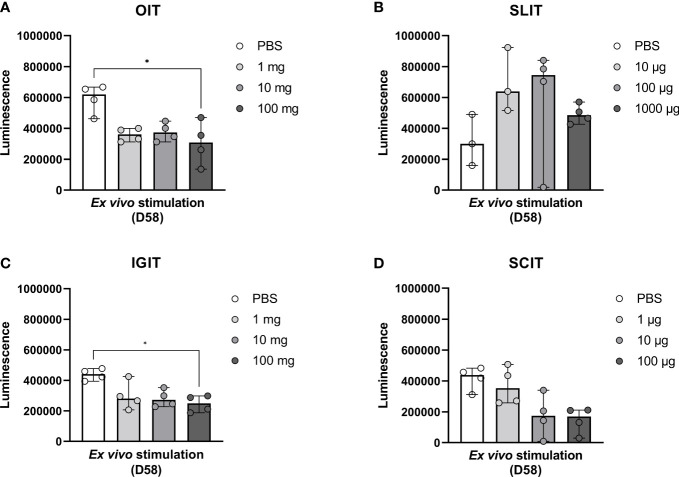
Peanut protein extract (PPE)-induced cellular responses upon stimulation of splenocytes with PPE. Animals were exposed to PPE by oral immunotherapy (OIT), sublingual (SL)IT, intragastric (IG)IT or subcutaneous (SC)IT and subsequently exposed to a weekly intraperitoneal (IP) injection for four weeks prior to sacrifice on Day 58. Spleens (n=4) were isolated from male animals of each dose group of **(A)** OIT, **(B)** SLIT, **(C)** IGIT and **(D)** SCIT including each control group administered with PBS. Splenocytes were stimulated with PPE in order to assess the degree of PPE-specific proliferation in each group for each IT. Median bars are presented for each group and each circle represents a single animal. Statistically significant differences compared to PBS administration are indicated with an asterisk, * p<0.05.

In order to assess the impact of OIT, SLIT, IGIT or SCIT on the PPE-induced cellular immune responses, splenocytes were stimulated with PPE to examine the *ex vivo* proliferative response. Splenocytes from high dosage groups of OIT and IGIT showed a statistically significant lower PPE-specific cellular response compared to splenocytes from control groups administered with PBS (**Figures 6A, C**). No statistically significant differences in PPE-induced proliferative response were seen for any of the dosage groups of SLIT or SCIT when compared to control groups administered with PBS (**Figures 6B, D**). These results support the PPE-specific IgE and EST results, stressing that rats administered with high doses of PPE OIT and IGIT mounted the lowest response upon exposure to PPE.

## 4 Discussion

Allergen-specific IT is an established treatment for respiratory allergies, yet allergen-specific IT remains mostly on an experimental level for treatment of food allergies where only one FDA-approved drug for treatment exists (Palforzia) (16, 25, 36, 37). Routes of administration, upscaling doses, maintenance dose, threshold level, age group and duration of therapy are some of the parameters that have been investigated in order to accommodate a favourable safety and efficacy profile for allergen-specific ITs for food allergies (25, 36). Yet, efficacy has often been reported to be at the expense of safety (3, 38, 39), which explains one of the challenges to develop adequate IT for food allergies. Considering that the prevalence of peanut allergy has increased substantially in recent decades (4, 6–8, 40), efforts to develop allergen-specific ITs targeting prevention of food allergies would be of high value. To accommodate this, animal models are important tools (32, 41) for facilitating the development of prophylactic strategies. Therefore, the present study aimed, to our knowledge for the first time, to investigate and compare in a dose-response relationship how PPE administered through different routes affected primary prevention of peanut allergy in a prophylactic BN rat model.

The present study showed that prevention of induction of peanut-specific IgE during the preventive regime, was dependent on both the dose and route of administration. High dose OIT as well as all doses of SLIT and IGIT did not induce sensitisation following the three weeks preventive regime. Contrary, low and medium doses of OIT as well as all doses of SCIT induced sensitisation. This demonstrated that a safe prevention regime devoid of sensitisation was compromised for certain doses of OIT and completely abolished upon all doses of SCIT, while a broader dose-range devoid of sensitisation was demonstrated for SLIT and IGIT. These findings favour SLIT and IGIT for potential use as future prophylactic ITs compared to OIT and SCIT. Indeed, the same dose (1 mg) induced sensitisation by oral route of administration, while no sensitisation was observed for SL and IG route of administration. A greater safety profile for SLIT compared to OIT has been reported previously (24, 42, 43), however doses applied in SLIT trials are up to 1000-fold lower than doses applied in OIT trials (20, 44), making a direct comparison between SLIT and OIT trials difficult. The fact that the same dosages of PPE applied for prophylactic OIT, SLIT and IGIT prompted large differences in the level of sensitisation clearly stresses that the sensitising capacity of peanut is dependent on the exposed mucosal site, and thus possibly how the peanut allergens are presented to the immune system. Thus, the present study revealed distinct patterns in the response to the allergen depending on allergen quantity and site of presentation to the immune system. This is in line with previous findings, showing that the route of administration determines the fate of the antigens, and thus the nature of the immune response (45). Furthermore, our findings are in accordance with studies on oral tolerance showing that differences in antigen dose regimes induce distinct immunological mechanisms (46).

We found that all SCIT doses were highly potent in inducing sensitisation, which indicates that the SCIT route may simply not be a suitable route for prevention of peanut allergy. However, the same doses of SCIT have been applied in a mouse model for treatment of peanut allergy showing clinical improvement of peanut allergy (26), thus suggesting that optimal doses for peanut specific SCIT are different for treatment and prevention of peanut allergy or between animal species.

Dose and route-dependent differences were also observed when assessing the level of protection from peanut allergy after OIT, SLIT, IGIT and SCIT. High dose OIT (100 mg) and SLIT (1 mg), as well as medium (10 mg) and high (100 mg) doses IGIT prevented IP-induced sensitisation to a statistically significant degree. More doses were shown effective after both IP injections and oral challenge, where high dose OIT (100 mg), medium (0.1 mg) and high (1 mg) doses SLIT, as well as low (1 mg), medium (10 mg) and high (100 mg) doses IGIT prevented the induction of specific IgE to a statistically significant degree. Thus, a broader dose-range of SLIT than OIT were effective in preventing peanut allergy, whereas an even broader dose-range was evident for IGIT.

The level of protection from IP-induced sensitisation was generally reflected in the clinical response to PPE in the EST. The clinical protection exerted by OIT and IGIT is in line with studies reporting a higher efficacy for OIT compared to SLIT (24). None of the dosage groups of SCIT prevented IP-induced sensitisation as all animals were already sensitised by the initial SCIT preventive regime.

In the *ex vivo* splenocyte proliferative response to PPE stimulation, high dose OIT and high dose IGIT were found to suppress proliferation to a statistically significant degree, in line with the exerted protection from IP-sensitisation. The proliferative response to PPE may though have been affected by the endotoxin content in the PPE, as it is well-acknowledged that endotoxin contamination may affect cell proliferative responses to allergens due to the immunostimulatory capacity (47, 48).

Interestingly, we found that 1 mg of PPE administered through the SL route was effective in preventing IP-induced sensitisation to a statistically significant degree, while 1 mg of PPE administered *via* IG route was only shown to be effective to a statistically significant degree after an oral challenge. Contrary, 1 mg PPE administered through the oral route did not prevent IP-induced sensitisation but instead promoted sensitisation. This suggests that this particular dose activated the immune system in a tolerogenic manner through the SL and IG routes as opposed to the oral route. The superior efficacy observed by SL administration of the 1 mg PPE could be explained by treatment studies showing that allergen-specific tolerance induced by SLIT is linked to a specific immune cell composition of the SL tissue, e.g. the limited number of proinflammatory cells such as mast cells and eosinophils and the tolerogenic properties of dendritic cells (DCs) such as Langerhans cells (49). Besides the protolerogenic immune milieu of the SL tissue, the induction of regulatory B cells and T cells secreting IL-10, IL-35 and TGF-β in lingual tonsils is linked to the mechanisms induced by SLIT in treatment settings as reviewed by Pinheiro-Rosa and co-workers (50). Further, the tolerance inducing capacity of PPE exerted through the oral and IG routes may also differ from each other and hence explain the different outcome of the 1 mg PPE in current study. The present study showed that a tolerogenic response could be induced by a lower allergen dose when bypassing the oral cavity by means of IGIT as opposed to engaging the oral mucosa by means of OIT. This indicates that targeted delivery to the gut promoted tolerance with lower PPE doses compared to OIT. Targeted delivery of peanut allergens to the gut is a novel strategy currently being investigated using nanoparticles for OIT (51). Nanoparticles for allergen-specific IT are constructed with the aim of protecting the loaded allergens from degradation by the harsh conditions of the gastrointestinal tract and facilitating the presentation of the allergens to the gut-associated lymphoid tissue to promote a tolerogenic response (51). On a further note, the IgG1-inducing ability of PPE through the OIT and IGIT routes were strikingly different. Whereas high dose OIT induced an IgG1 response after the three weeks preventive regime, high dose IGIT did not. However, both high dose OIT or IGIT prevented IP-induced sensitisation and PPE-induced elicitation, revealing that prevention for peanut allergy may be driven by distinct immunological mechanisms for OIT and IGIT. A blocking effect exerted by allergen-specific IgG1 may be the driving force of high dose OIT in preventing IP-induced sensitisation. It has been reported that OIT for treatment of peanut allergy induces increased antigen-specific IgG that exerts blocking abilities and promotes desensitisation in peanut allergic individuals (52). Low and medium doses OIT exerted a greater allergen-specific IgG1 response than the high dose and led to development of peanut allergy. Thus, avoiding the development of allergen-specific IgG1 may be a more safe pathway for tolerance induction as evidence suggests that IgE cells primarily originate from antigen-experienced IgG1-expressing cells (53). Therefore, IGIT may be a more favourable strategy for tolerance induction compared to OIT. IL-4, IL-5 and IL-13 secreted by Th2 cells promote generation of antigen-specific IgG1 and IgE from activated B cells (20), hence a suppressive mode of action by inhibition of Th2 differentiation and the cytokines thereof could explain the suppression and lack of antigen-specific IgG1 response seen for high dose OIT and IGIT, respectively. It is known that regulatory T cells can suppress responses of effector T cells such as Th2 cells by the action of IL-10 and TGF-β (54), and adoptive transfer of antigen-specific regulatory T cells have shown to suppress food allergy in a murine model (55), thus demonstrating the capability of this T cell subset to counteract unfavourable antigen-specific immune responses. Moreover, regulatory T cells express different chemokine receptors as a consequence of their initial tissue site of priming, thus different routes of allergen-specific IT can lead to regulatory T cells with different homing properties and thereby distinct efficacies *in vivo* (33).

As the four major peanut allergens Ara h 1, Ara h 2, Ara h 3 and Ara h 6 have been recognised by serum IgE in 45-90% of peanut-sensitised individuals (56–59), prevention should be targeted primarily towards these allergens. Further, Ara h 2 and Ara h 6 have been reported to be the major elicitors of effector cell degranulation (60), thus emphasising the importance of preventing sensitisation to these allergens. Ara h 3 was present in PPE in a higher amount than any other of the major allergens, similar to what have been found in other early peanut intervention products (61), whereas Ara h 1, Ara h 2 and Ara h 6 were found in lower and similar quantities. Yet, Ara h 1, Ara h 2, Ara h 3 and Ara h 6-specific IgE levels were all found to follow PPE-specific IgE levels. This finding indicates that no specific major allergen in PPE drives the sensitising or preventive capacity of the PPE. Instead, other underlying factors seem to affect the efficacies of the prophylactic ITs observed here. Capture of antigen by antigen presenting cells such as DCs is required for the induction of a T cell response and subsequent Ig response. The phenotype of DCs are tissue-specific and determines the fate of the naïve T cell and its homing properties (62). Hence, it seems reasonable to assume that different phenotypic DCs come into play depending on the route of administration, thereby resulting in different T cell responses which may or may not lead to Ig responses. It has been shown that OIT and SLIT induce different immunomodulatory effects on myeloid and plasmacytoid DCs in blood, which in turn influenced the resulting T cell response (63). In addition, OIT for treatment of peanut allergy has shown to alter the phenotype of DCs and thus facilitate the induction of regulatory T cells (64).

To conclude, the present study showed that the preventive effect of allergen-specific IT is determined by the interplay between i) the specific site of PPE delivery for presentation to the immune system, and ii) the allergen quantity. These findings warrant further investigations with a focus on determining the underlying immunological mechanisms that drive the response to a route-specific prophylactic IT. Such investigations are currently ongoing with a focus on e.g. determining a more detailed Ig profiling (IgG2a, IgG2b, IgG2c and IgA) in serum, cytokine profiling of stimulated splenocytes, gene expression profiling in intestinal tissues and protein intestinal uptake analyses in BN rats.

## Data availability statement

The MS/MS datasets presented in this study can be found in online repositories. The names of the repository/repositories and accession number(s) can be found here: PXD039324 (PRIDE). Other data supporting the findings of this study are available from the corresponding author upon reasonable request.

## Ethics statement

The animal study was reviewed and approved by the Danish Animal Experiments Inspectorate with the authorisation number 2015-15-0201-00553-C1. The animal experiment was overseen by the National Food Institute’s in-house Animal Welfare Committee for animal care and use.

## Author contributions

KB and JL conceived of the study. NR and SK prepared PPE and individual allergens. TS performed SDS-PAGE and SEC and JM performed MS/MS analyses. TS and LN-N performed ELISAs. N-PK performed *ex vivo* stimulation experiment. KB, JL, AS, PJ, AM and SG supervised analyses. TS presented the results and drafted the manuscript. JL and KB revised the manuscript. All authors made substantial intellectual contributions, reviewed the manuscript critically, and approved the final version of the manuscript.
