# Supplementary material for: Dose and route of administration determine the efficacy of prophylactic immunotherapy for peanut allergy in a Brown Norway rat model
Source: Front Immunol. 2023 Feb 23;14:1121497. doi: 10.3389/fimmu.2023.1121497 (PMC9996042; doi:10.3389/fimmu.2023.1121497)
Supplement: Supplementary file 1 [file DataSheet_1.pdf]

Supplementary Material

1. Supplementary MATERIALS AND METHODS

1.1. Purification of peanut protein extract, Ara h 1, Ara h 2, Ara h 3 and Ara h 6

1.1.1. Preparation of peanut protein extract (PPE)

PPE was prepared from defatted peanut flour. Initially, de-skinned raw peanuts (Morrisons market, Leeds, UK) were ground and extracted with hexane (1:5, w/v hexane to flour), grounding and defatting repeated, and subsequently stirred in milli-Q (MQ) water for 1 h (10:1, v/w MQ water to flour). The pH of the extraction mixture was maintained at 10 by the addition of 1 M NaOH as required. The solubilised protein was collected by centrifugation (3000*g* for 20 min, 20ºC). The pH of the solubilised protein supernatant was adjusted to pH 4.5 with 1 M HCl and allowed to stand for 1 h at 20ºC. The precipitated protein was collected by centrifugation (3000*g*, 5 min, 20ºC) and subsequently washed twice by resuspension, mixing and centrifugation (3000*g,* 5 min, 20ºC) with two volumes of MQ water (pH 4.5, adjusted with 1 M HCl). The resulting pelleted material was freeze dried and finely ground to produce the PPE.

1.1.2. Purification of Ara h 1

Briefly, raw peanuts were peeled, ground and defatted with hexane (5:1, w/v), re-ground and the defatting repeated to obtain defatted peanut flour. Defatted peanut flour was extracted by stirring with water (1:5, w/v) for 1 h at 20°C. The extract was centrifuged (3000*g*, 15 min, 20°C) and the supernatant collected and centrifuged (30,000*g*, 40 min, 20°C). The solubilised protein was subjected to sequential ammonium sulfate fractionation and the protein fraction soluble between 70 and 100% ammonium sulfate saturation at 20°C was collected by centrifugation (3000*g*, 30 min, 20°C). The pelleted material was redissolved in 20 mM Tris, 500 mM NaCl, pH 7.5 before being dialysed against this buffer overnight (10-12 kDa). After clarification by centrifugation (30,000*g*, 40 min, 20°C) and filtration (0.2 µm, regenerated cellulose), portions (50 mL) were loaded at 1 mL/min onto a column of Con A Sepharose (1.6 x 10 cm; GE Healthcare, Amersham, UK) attached to ÄKTAPurifier FPLC system (GE Healthcare). Unbound protein was eluted with fresh dialysis buffer at 1.5 mL/min before bound protein was eluted with dialysis buffer containing 0.4 M methyl manopyranoside (Sigma, Gillingham, UK). The absorbance of the eluate was monitored at 280 nm and protein containing fractions were analysed by SDS-PAGE and those containing pure Ara h 1 were pooled, dialysed exhaustively against 20 mM ammonium bicarbonate, pH 7.0, freeze dried and stored frozen.

1.1.3. Purification of Ara h 2 and Ara h 6

Briefly, raw peanuts were peeled, ground and defatted with hexane (5:1, w/w), re-ground and the defatting repeated to obtain defatted peanut flour. Subsequently, proteins were extracted using 50 mM Tris/HCl buffer (pH 7.4), (1:7.5, w/v) with stirring for 1 h at 20°C. The protein suspension was centrifuged (3000*g*, 30 min, 20°C) and the supernatant collected. The solubilised protein was subjected to sequential ammonium sulfate fractionation and the protein fraction soluble between 50 and 75% ammonium sulfate saturation at 20°C was collected by centrifugation (3000*g*, 30 min, 20°C). The pelleted material was resuspended as a 5% solution (w/v) in 20 mM Tris/HCl, 150 mM NaCl, pH 7.4, and after filtration (0.22 µm), 1.5 mL portions of the crude extract were loaded onto a Superdex-75 column (1.6 x 65 cm; GE Healthcare) equilibrated with the same buffer, attached to ÄKTAPurifier FPLC system (GE Healthcare). The column was eluted at 1 mL/min for 1.2 column volume (CV). The absorbance of the eluate was monitored at 280 nm and the fractions collected were analysed by SDS-

PAGE and reverse phase HPLC. Ara h 2 enriched fractions were pooled, and the ammonium sulfate concentration of the solution was adjusted to 2 M. The solution was filtered through a 0.22 µm filter and then subjected to hydrophobic interaction chromatography for further purification.

The protein solution was loaded onto a Phenyl Sepharose High Performance, column (1.6 x 16 cm; GE Healthcare) attached to an ÄKTAPurifier FPLC system, equilibrated with 25 mM Tris/HCl buffer containing 2 M ammonium sulfate, pH 7.0. After the elution of unbound material, the bound proteins were eluted with a decreasing gradient of ammonium sulfate, (2 M to 1.4 M over 1 CV, 1.4 M to 0.8 M over 17 CVs, 0.4 to 0.0 M over 1 CV) and the fractions collected were analysed by SDS-PAGE and reverse phase HPLC. Fractions containing purified Ara h 2 or Ara h 6 were adjusted to 2 M ammonium sulfate and reapplied to the above column equilibrated in the above buffer. As soon as the protein solution was loaded, the column was eluted with a step gradient of the above buffer containing no ammonium sulfate. The concentrated protein solutions were firstly concentrated further using a stirred ultrafiltration cell (Millipore UFSC400, fitted with a 3 kDa ultrafiltration regenerated cellulose membrane). Following the concentration stage, samples were desalted by repeated dilution and re-concentration with water. After a final concentration step by ultrafiltration the samples were freeze dried and stored with 50 mM ammonium bicarbonate, pH 7.0. Solution aliquots of 10 mL were loaded at a flow rate of 1 mL/min, then the column was eluted with 50 mM ammonium bicarbonate at a flow rate of 4 mL/min and a gradient length of 0.9 CV. The absorbance of the eluate was monitored at 280 nm, and by conductivity the desalted protein rich fraction was freeze dried and stored at -25°C until use.

1.1.4. Purification of Ara h 3
Ara h 3 was purified from defatted peanut flour prepared from raw peanut, essentially as described earlier (1). After anion exchange column (Source Q operated on ÄKTAPurifier FPLC system, GE Healthcare), Ara h 3 was found in fractions eluting at approximately 400 to 500 mM NaCl. Only fractions with no other visible bands than described for Ara h 3 (2) were used. Ara h 3 was stored lyophilised and refrigerated.

1.2. MS/MS analysis

The lyophilised PPE sample was re-suspended in PBS (137 mM NaCl, 3 mM KCl, 8 mM Na_2HPO_4, 1 mM KH_2PO_4, pH 7.2) at 2.4 µg/µL. Fifty microliters of this sample was precipitated overnight at -20°C in 250 µL of ethanol/acetone/formic acid at a 49.95: 49.95:0.1 ratio. The resultant pellet was washed separately in 100% (v/v) acetone and 75% (v/v) ethanol and dried by centrifugal evaporation. The pellet was reconstituted in 180 µL of 50 mM ammonium bicarbonate, 126 µL water and 18 µL of 100 mM dithiothreitol and heated to 95°C, for 5 min. The protein was alkylated by adding 36 µL of 100 mM of iodoacetamide and incubated at room temperature (RT) in the dark for 20 min. The sample was digested by adding 12 µL of 100 ng/µL Pierce Trypsin Protease, MS grade (90059, Thermo Scientific) and incubated at 37°C for 1 h. A further 12 µL of 100 ng/µL Trypsin was added and samples incubated at 30°C overnight. Ninety-six µL of the digested sample (equivalent to 30 µg of the PPE sample) was eluted from a Pierce C18 spin column (89870, Thermo Scientific) according to manufacturer's instructions. Samples were frozen at -80°C prior to lyophilisation by centrifugal evaporation and the dried samples were resuspended in 30 µL of 5% (v/v) acetonitrile, 0.1% (v/v) formic acid.

One-dimensional (1D) microscale liquid chromatography separation of tryptic peptides (2 µL injection) was performed, in duplicate (2 technical replicates), with an UltiMate 3000 RSL® liquid chromatography (UPLC) system (IQLAAAGABHFAPBMBEZ, Thermo Scientific), equipped with a Hypersil Gold C18 1.9 µm, 100 × 1 mm analytical reversed phase column (Thermo Scientific). Mobile phase A consisted of water containing 0.1% (v/v) formic acid, whilst mobile phase B was 100% (v/v) acetonitrile containing 0.1% (v/v) formic acid. The sample was injected on-column and peptides were eluted from the analytical column and separated using a gradient of 2–40% mobile phase B over 60 min at a flow rate of 60 µL/min. The analytical column temperature was maintained at 35°C.

Mass spectrometric analysis utilised a Q Exactive Plus™ Hybrid Quadrupole-Orbitrap™ MS (Thermo Scientific) in the data-dependent acquisition mode with survey scans acquired at a resolution of 70,000 at m/z 400, whereas the target value for the fragment ion spectra was set to resolution of 17,500 at m/z 400. Up to the top 10 most abundant isotope patterns with charge 2 to 4 from the survey scan were selected with an isolation window of 1.5 Thomsons and fragmented by higher energy collisional dissociation with normalised collision energies of 27. The maximum ion injection times for the survey scan and the MS/MS scans were 100 and 60 ms, respectively, and the ion target value for scan modes were set to 1E6 and 2E5, respectively. Repeat sequencing of peptides was kept to a minimum by dynamic exclusion of the sequenced peptides for 10 s.

Peaks version 8.5 software was used to process all data-dependent acquisition mass spectral data. Protein identifications were obtained by searching a database of peanut allergens and isoforms (outlined in Apostolovic et al. (3)) and the downloaded reference proteome of peanut. The following data analysis parameters were used: tryptic rules; no mis-cleavages; carbamidomethylation as a fixed modification and oxidation of hydroxyproline as a variable modification; parent mass error tolerance of 2 ppm, fragment mass error tolerance of 0.05 Da; FDR of 1%; charge states of +2, +3 and +4 accepted. Table S1 shows the protein coverage, -Lg10P, number pf peptides detected for each protein identified.

1.3. Animal experimental design

PBS (as control) or PPE in low, medium and high doses were administered through four different routes: oral, intragastric (IG), sublingual (SL), and subcutaneous (SC) administration. PPE administration through oral route was performed by dosing the rats in the oral cavity while allowing the animals to swallow the PPE solution meanwhile. SL PPE administration was performed by holding the animal by the scruff, devoid of swallowing opportunity, and carefully applying a droplet of the PPE solution under the tongue. The animals were held by the scruff for an additional 20 s to retain the drop under the tongue and to avoid swallowing. Whereas oral and SL PPE administration enabled contact between the PPE solution and the oral mucosa, IG PPE administration bypassed the oral mucosa for direct delivery of the PPE in the stomach. Oral, SL and IG administrations were performed daily. SC PPE administration was performed by SC injection in the abdominal skin three times per week (Monday, Wednesday, Friday).

1.4. ELISA – quantification of antibody titres

IgG1 and IgE specific for PPE, Ara h 1, Ara h 2, Ara h 3 and Ara h 6 were quantified by means of indirect and antibody-capture ELISA, respectively.

IgG1 ELISA was performed by coating plates (96 well, microtitre, Maxisorp, Nunc, Roskilde, Denmark) with 100 µL of 0.5 µg/mL of PPE, Ara h 1, Ara h 2, Ara h 3 or Ara h 6 in carbonate buffer (15 mM Na_2CO_3, 35 mM $NaHCO_3$, pH 9.6) and incubating overnight at 4°C. Plates were washed five

times in PBS with 0.01% (w/v) Tween 20 (P1379, Sigma-Aldrich) (PBS-T) after each incubation step. Subsequently, 50 µL/well of two-fold serial dilutions of serum and positive and negative control samples diluted in PBS-T (starting at 1:8) were added, and plates were incubated for 1 h at RT. For detection, 50 µL/well of HRP-labelled mouse-anti-rat-IgG1 (3060-05, Southern Biotech, Birmingham, AL, US) diluted 1:20,000 (v/v) in PBS-T was added, and plates were incubated for 1 h at RT. To visualise the enzymatic reaction, 100 µL/well of 3,3',5,5-tetramethylbenzidine (TMB)-one substrate was added, and plates were incubated for 12 min at RT. The reaction was stopped by adding 100 µL/well of 0.2 M H_2SO_4 and absorbance was measured immediately after at 450 nm with a reference wavelength of 630 nm using a microtitre reader (Gen5, BioTek Instruments, Winooski, VT, US).

IgE ELISA was performed by coating plates (96 well, Maxisorp, Nunc) with 100 µL/well of 0.5 µg/mL mouse anti-rat (HDMAB-123 HybriDomus, Cytotek, Hellebæk, Denmark) in carbonate buffer followed by incubating plates overnight at 4°C. Plates were washed five times with PBS-T between each incubation step. To block unspecific binding, 200 µL/well of 3% (v/v) rabbit sera (S2500-500, Biowest, Nuaillé, France) in PBS-T was added, and plates were incubated for 1 h at 37°C. Subsequently, 50 µL/well of two-fold serial dilutions of serum and positive and negative control samples diluted in PBS-T (starting at 1:8) were added, and plates were incubated for 1 h at RT. Next, 50 µL/well of 0.2 µg/mL of digoxigenin (DIG)-coupled PPE (10:1), 0.2 µg/mL DIG-Ara h 1 (10:1), 0.2 µg/mL DIG-Ara h 3 (10:1) or 0.1 µg/mL DIG-Ara h 6 (10:1) in 3% (v/v) rabbit sera (Biowest) in PBS-T was added to the plates and incubated for 1 h at RT. For detection of DIG-coupled PPE or individual allergens bound to IgE, 50 µL/well of HRP-labelled sheep-anti-digoxigenin (Anti-Digoxigenin-POD 1633716001, Roche Diagnostics GmbH, Mannheim, Germany) diluted 1:1,000 (v/v) in PBS-T was added and plates were incubated for 1 h at RT. Visualisation was performed as described for IgG1 ELISAs.

Antibody titres are expressed as $\log_2$ titre values and defined as the interpolated dilution of the given serum sample leading to the mean absorbance for the negative control serum + 3 standard deviation (SD).

1.5. *Ex vivo* spleen proliferation

Spleens (n=4/group) from male control and treated rats were removed after sacrifice (Day 58) and placed in 10 mL cold sterile PBS on ice. Spleens were transferred to gentleMACS C tubes (130-093-237, Miltenyi Biotec, North Rhine-Westphalia, Germany) in 5 mL cold sterile PBS and placed in a gentleMACs dissociator (130-093-235, Miltenyi Biotec) to allow automated tissue dissociation. Tissue samples were transferred to 15 mL tubes and centrifuged (400*g,* 5 min, 4°C). The supernatant was discarded, and the pellet was resuspended in 1 mL cold sterile PBS and transferred to a 50 mL tube through a 70 µm cell strainer (352350, Corning Incorporated, Kennebunk, ME, US). Cold sterile PBS was added to the resuspension up to 20 mL and centrifuged (400*g*, 5 min, 4°C). The supernatant was discarded, and the pellet was agitated by grinding the bottom of the tube on a laminar air flow grill. Finally, splenocyte single cell suspensions were obtained by suspending the pellet in 5 mL cold RPMI 1640 medium (72400-021, Thermo Scientific) with 5% (v/v) FBS (10500-064, Thermo Scientific) and 0.1% (v/v) [50 µg/mL] Gentamycin (15750-037, Thermo Scientific), 0.15 mM [16.6 µg/mL] monothioglycerol (M6145, Sigma). Splenocytes were counted on a Sysmex XT-1800i and 4.0 x 10^5 cells were added to each well of a 96-well flat-bottom culture plate (3917, Corning Incorporated) and stimulated with 50 µg PPE, medium as negative control or 0.02 µg/mL PMA/0.4 µg/mL ionomycin as positive control. Stimulated cells were incubated for 72 h. The number of live splenocytes was determined by absorbance quantification measured by Glomax proliferation CellTiter-Glo (Promega, Madison, WI, US). Due to the assay being very time-consuming when analysing responses from

individual spleens, only splenocytes from one gender were collected to be able to perform the assay in one day and hence allowing the sacrifice and termination of the studies within the same day. Choosing male spleens over female spleens attribute to male spleens being much larger than female spleens.

1.6. Supplementary References

1. Koppelman SJ, Knol EF, Vlooswijk RAA, Wensing M, Knulst AC, Hefle SL, et al. Peanut allergen Ara h 3: Isolation from peanuts and biochemical characterization. Allergy [Internet]. 2003 Nov 1 [cited 2022 Jan 27];58(11):1144–51. Available from: https://onlinelibrary.wiley.com/doi/full/10.1034/j.1398-9995.2003.00259.x
2. Piersma SR, Gaspari M, Hefle SL, Koppelman SJ. Proteolytic processing of the peanut allergen Ara h 3. Mol Nutr Food Res. 2005;49(8):744–55.
3. Apostolovic D, Marsh JT, Baumert J, Taylor SL, Westphal A, de Jongh H, et al. Purification and Initial Characterization of Ara h 7, a Peanut Allergen from the 2S Albumin Protein Family. J Agric Food Chem [Internet]. 2021 [cited 2022 May 2];69:6318–29. Available from: https://doi.org/10.1021/acs.jafc.1c00618

2. Supplementary TABLES

TABLE S1: Origin and characteristics of the detected allergens in the PPE sample.
Protein origin (NCBI ID or gene position) are listed. Tryptic peptide coverage sequence is shown, including number of total peptides and number of unique peptides. -10LogP probability scores reported by PEAKS v8.5 indicates the scoring significance of a peptide-spectrum match.

Protein ID or gene position (NCBI)	**Allergen (description as per Apostolovic et al** (3)**)**	-10lgP	Tryptic peptide coverage (%)	#Total Peptides	#Unique peptides	#Spectral counts
NP_001363136.1	Arah1_1_09	307.28	51	27	9	161
NP_001363139.1	Arah1_2_19	306.67	47	24	6	157
NP_001363146.1	Arah2_1_08	192.6	63	11	5	80
XP_025671514.1	Arah2_2_18	195.18	65	11	5	80
XP_025644723.1	Arah3_1_02	224.03	31	14	1	50
XP_025650075.1<br>XP_025633145.1	Arah3_2_02/<br>Arah3_12_12	71.36	14	4	4	7
XP_025603245.1<br>XP_025607222.1	Arah3_3_06/<br>Arah3_6_06	210.34	42	11	1	36
XP_025603242.1<br>XP_025603240.1	Arah3_4_06/<br>Arah3_5_06	345.71	63	23	7	210
XP_025603243.1	Arah3_7_06	291.81	43	16	4	131
XP_025603244.1<br>XP_025663188.1	Arah3_8_06/<br>Arah3_15_16	156.16	26	9	8	25
XP_025603268.1	Arah3_9_06	162.75	43	13	2	22
XP_025603315.1	Arah3_10_06	345.79	63	25	7	208
XP_025632438.1	Arah3_11_12	221.65	31	14	1	47
XP_025663039.1	Arah3_13_16	305.95	51	18	1	166
XP_025663119.1	Arah3_14_16	163.93	43	13	2	23
XR_003179792.1	Arah3_16_16	318.06	57	20	7	162
XP_025663190.1<br>XP_025663194.1	Arah3_17_16/<br>Arah3_20_16	328.95	63	24	11	190
XP_025663195.1	Arah3_18_16	158.14	22	7	6	19
NP_001363142.1	Arah3_19_16	181.31	32	11	1	33
XP_025614701.1	Arah6_1_08	204.18	58	7	1	79
NP_001363141.1	Arah6_2_18	215.17	58	7	1	83
XR_003156017.2	Arah7_1_08	181.51	41	6	1	26
XR_003187481.2	Arah7_2_18	161.22	41	6	1	25

3. Supplementary FIGURES

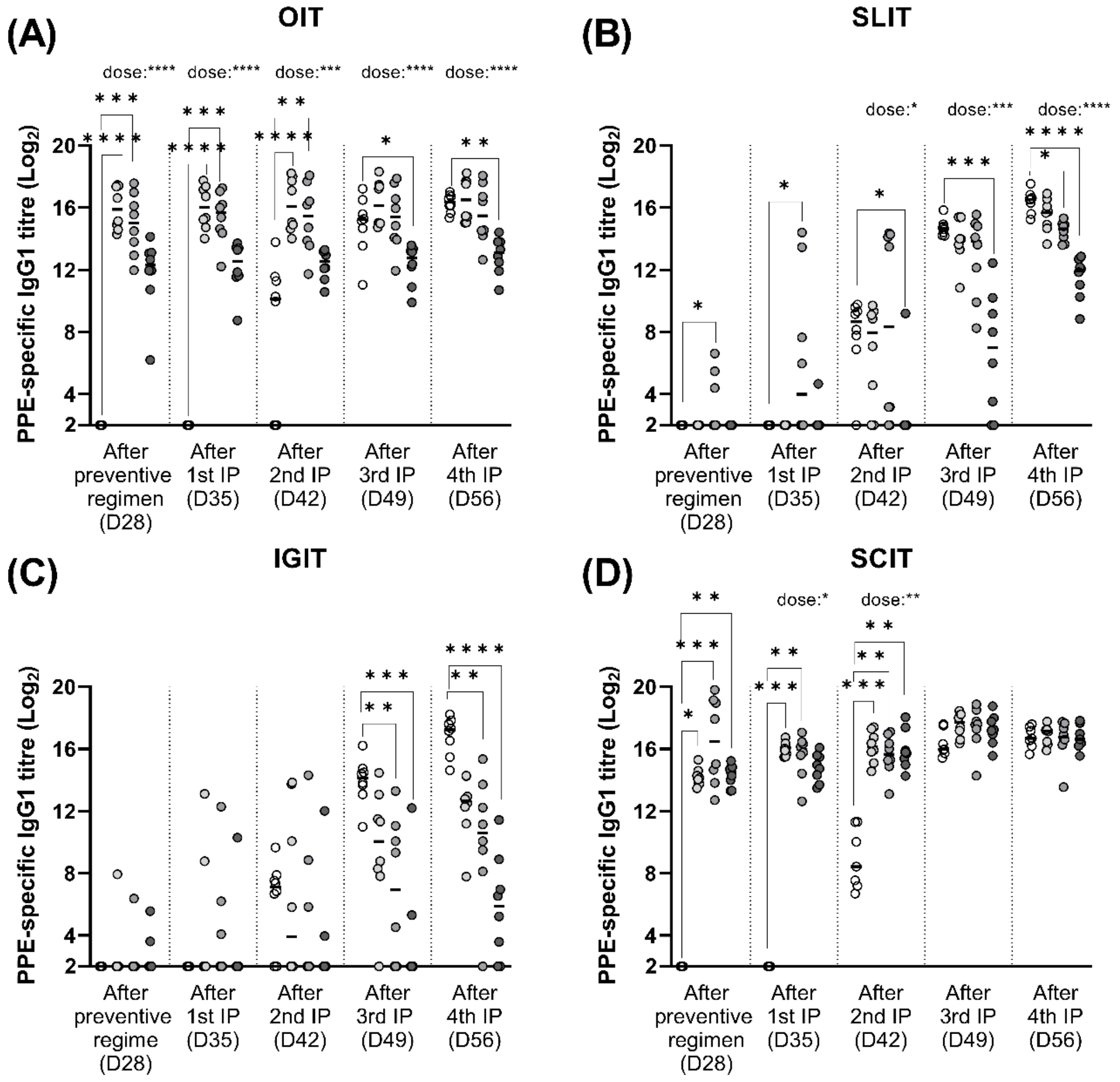

Supplementary FIGURE 1. The progression on peanut protein extract (PPE)-specific IgG1 from day (D) 28-56. PPE-specific IgG1 was determined in rat sera after the preventive regimen with either (A) oral (O) immunotherapy (IT), (B) sublingual (SL)IT, (C) intragastric (IG)IT or (D) subcutaneous (SC)IT, as well as after each intraperitoneal (IP) post-immunisation. The order of groups from left to right is for each day and each preventive regime; PBS, low dose, medium dose and high dose. Each symbol depicts the level of an individual animal and horizontal lines indicate median values in each group. Statistically significant differences compared to control animals receiving PBS administration are indicated with asterisk(s), * p<0.05, ** p<0.01, *** p<0.001, **** p<0.0001.

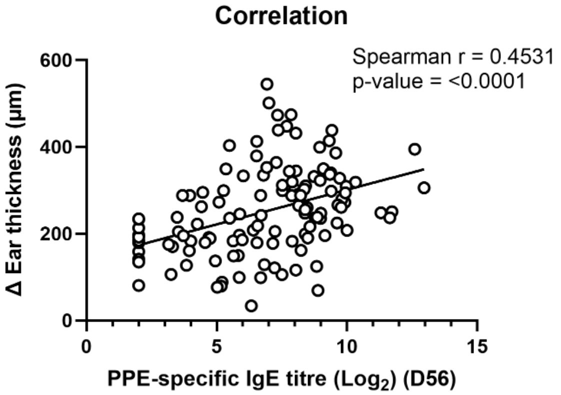

Supplementary FIGURE 2. Correlation between peanut protein extract (PPE)-specific IgE at Day 56 and delta ear swelling. Each symbol represents an individual rat. Non-parametric Spearman correlation was calculated PPE-specific IgE and delta ear swelling.

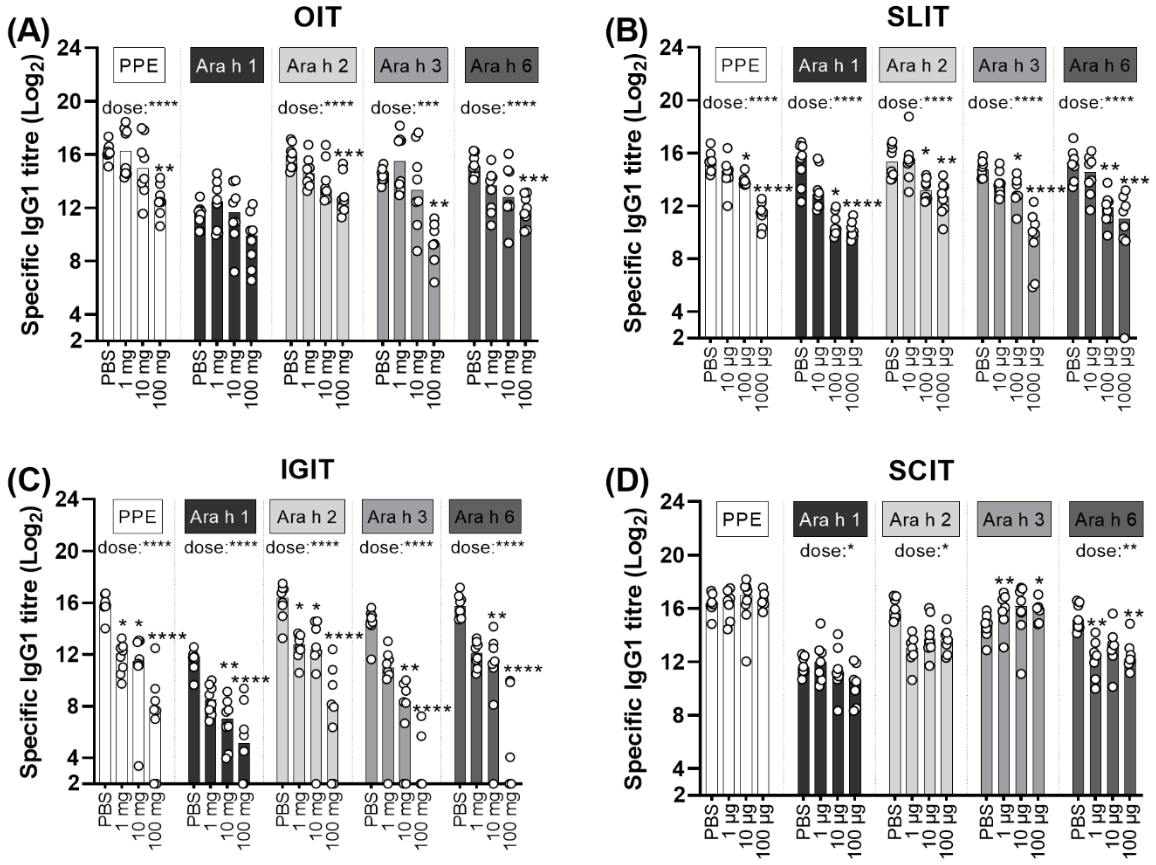

Supplementary FIGURE 3. Allergen-specific IgG1 at sacrifice. Specific IgG1 titres determined in sera of rats exposed to (A) oral (O) immunotherapy (IT), (B) sublingual (SL)IT, (C) intragastric (IG)IT, or (D) subcutaneous (SC)IT. Levels of peanut protein extract (PPE), Ara h 1, Ara h 2, Ara h 3 and Ara h 6-specific IgG1 were quantified by ELISA in sera from the day of sacrifice (D58). Median bars are

presented for each group and each symbol represents a single animal. The level of allergen-specific IgG1 is the sum of a three week's preventive regime (Day 0-20) with OIT, SLIT, IGIT or SCIT with three different doses of PPE or PBS followed by four intraperitoneal (IP) injections with PPE (Day 28, 35, 42 and 49) for assessing the efficacy of each IT. Statistically significant differences compared to control animals receiving PBS administration are indicated with asterisk(s), * $p<0.05$, ** $p<0.01$, *** $p<0.001$, **** $p<0.0001$.
